# Supplementary material for: Fluorescence-based thermal sensing with elastic organic crystals
Source: Nat Commun. 2022 Sep 8;13:5280. doi: 10.1038/s41467-022-32894-w (PMC9458730; doi:10.1038/s41467-022-32894-w)
Supplement: Supplementary file 1 — Supplementary Information [file 41467_2022_32894_MOESM1_ESM.pdf]

SUPPLEMENTARY INFORMATION

Fluorescence-Based Thermal Sensing with Elastic Organic Crystals

Di et al.

Supplementary Figures

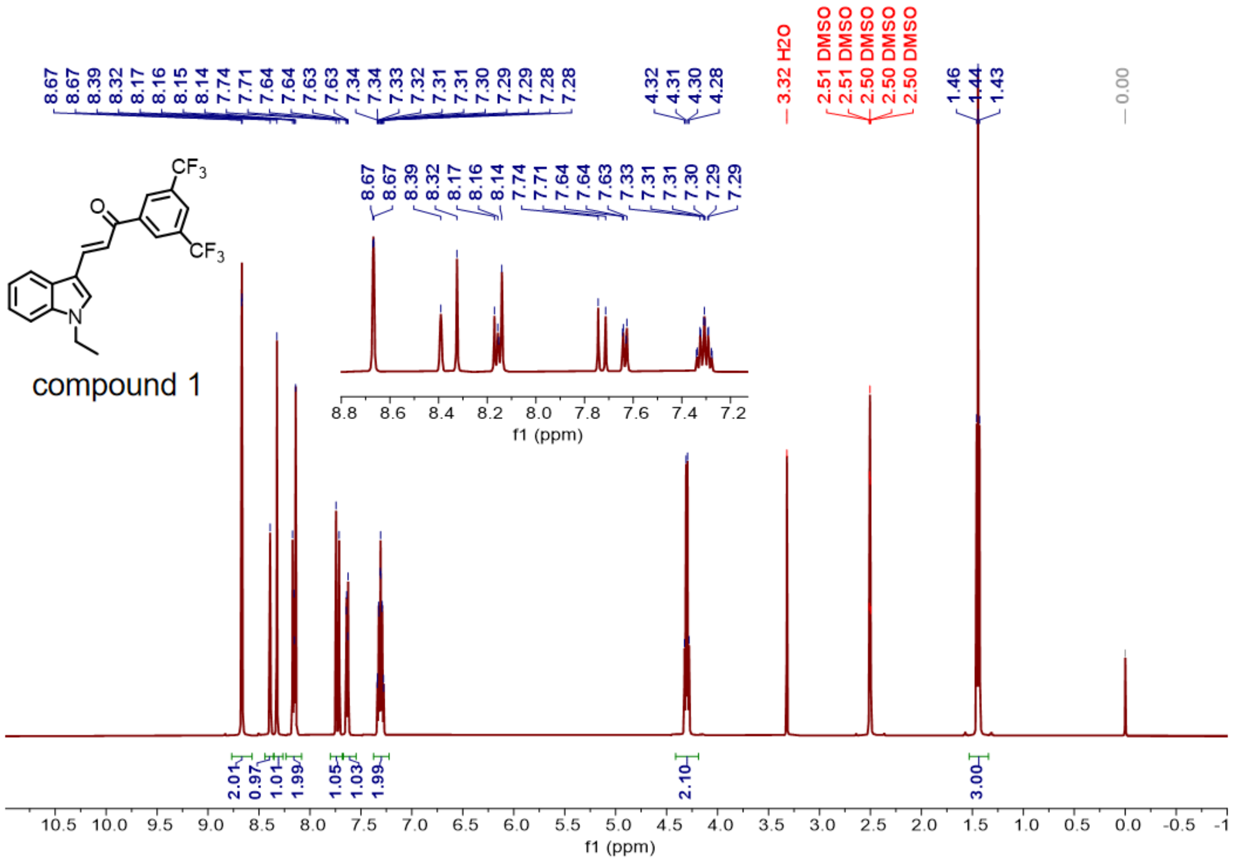

Supplementary Figure 1. ^{1}H NMR spectrum of compound 1 (500 MHz, DMSO-d_6).

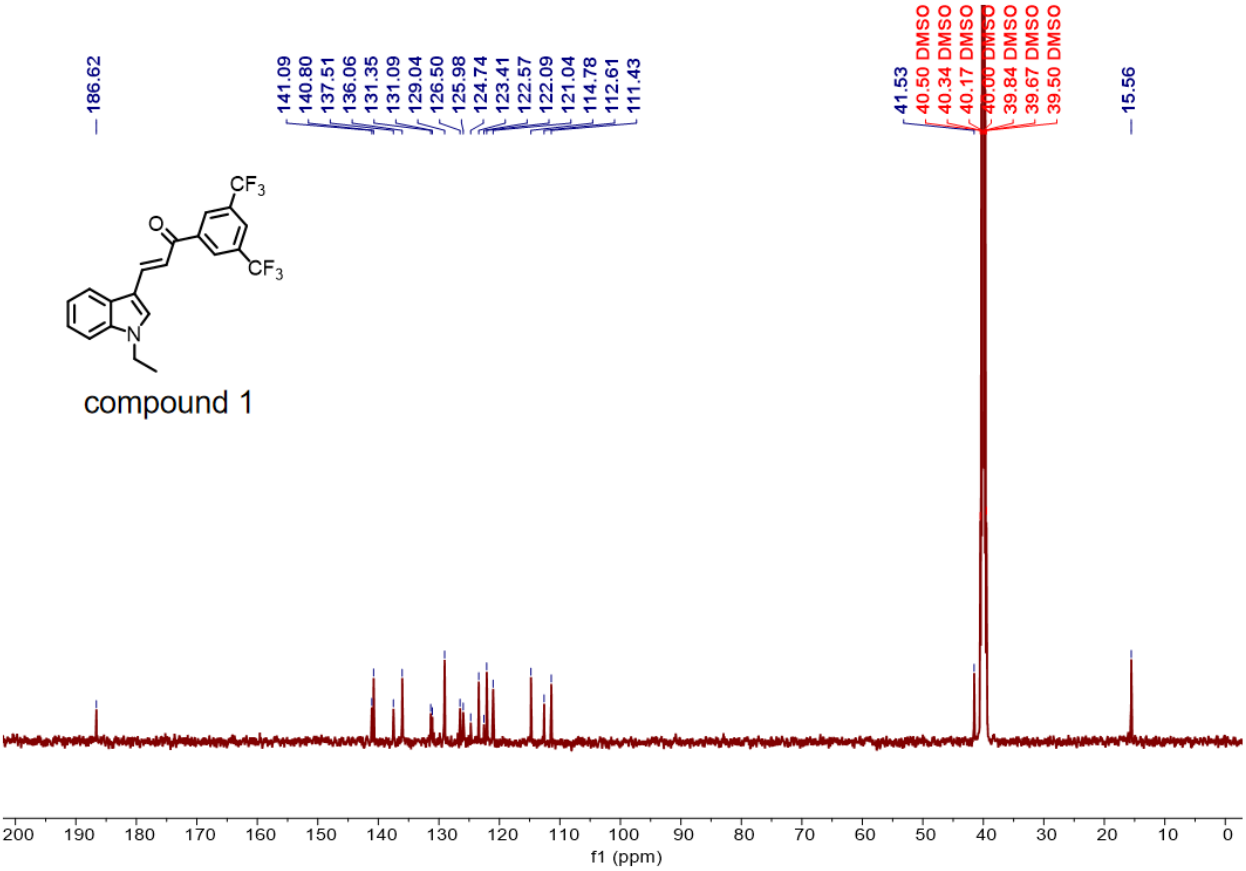

Supplementary Figure 2. ^{13}C NMR spectrum of compound 1 (126 MHz, DMSO-d_6).

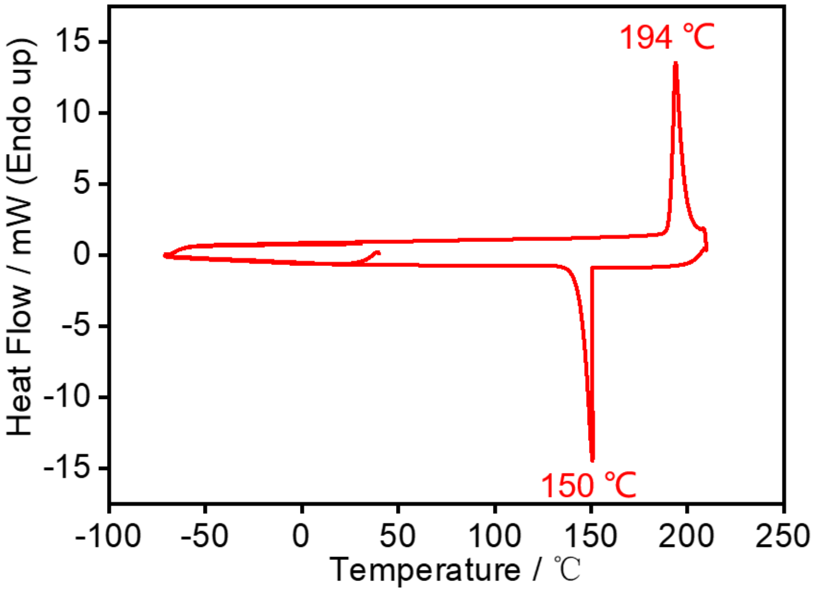

Supplementary Figure 3. Differential Scanning Calorimetric (DSC) analysis of crystals of compound 1 recorded at heating/cooling rate of 20 K/min.

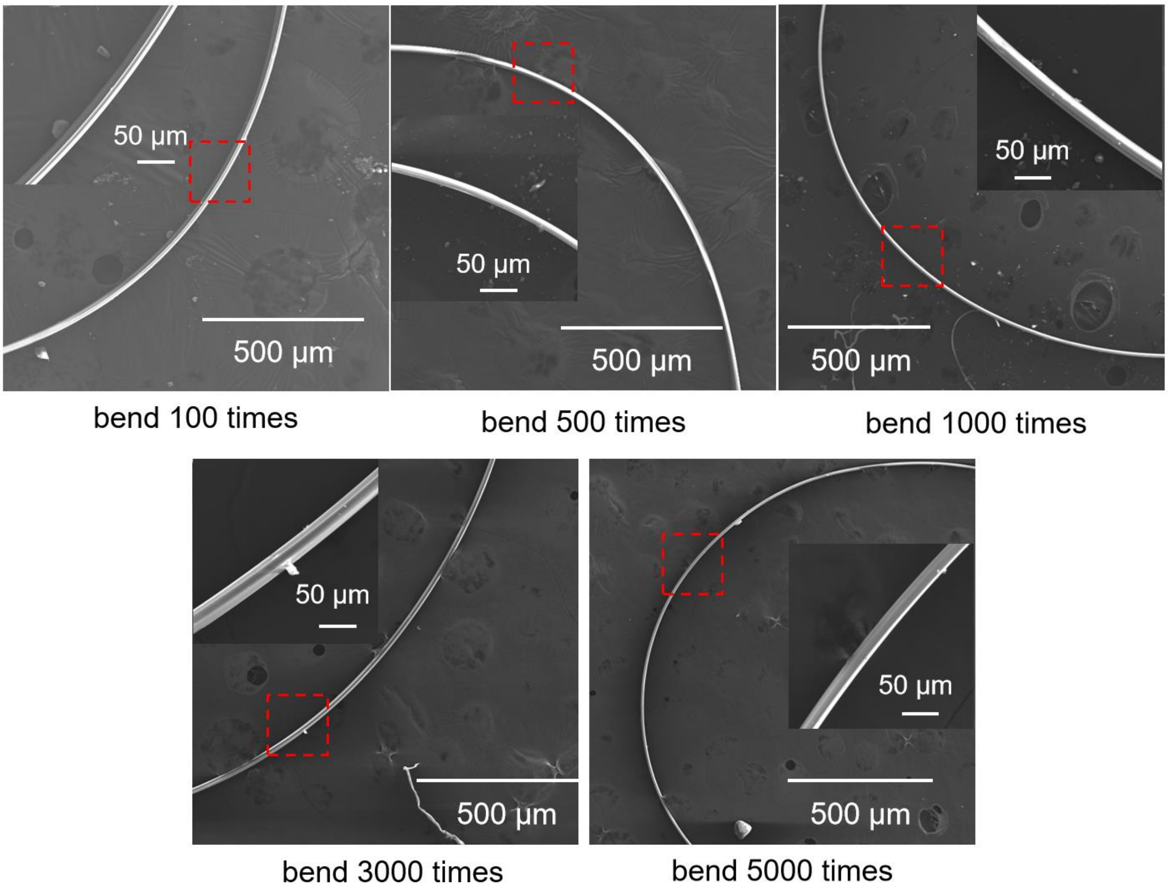

Supplementary Figure 4. SEM images of five different crystals of compound 1 after they were bent 100, 500, 1000, 3000, and 5000 times.

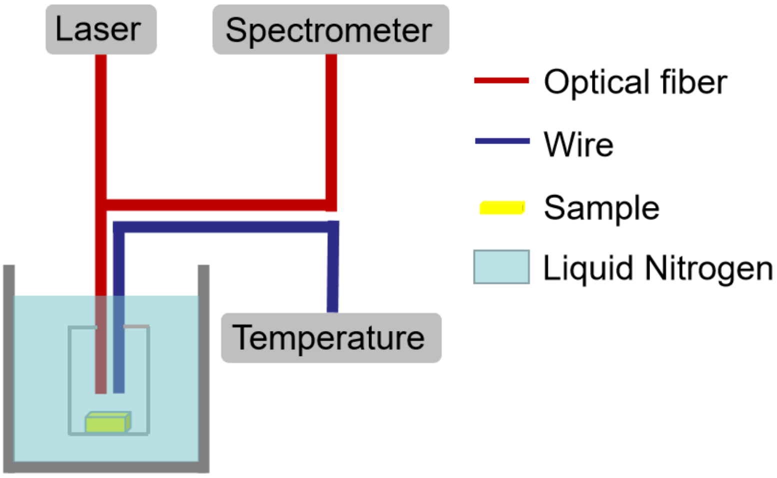

Supplementary Figure 5. Diagram of the experimental setup used for the temperature-dependent fluorescence spectroscopic measurements.

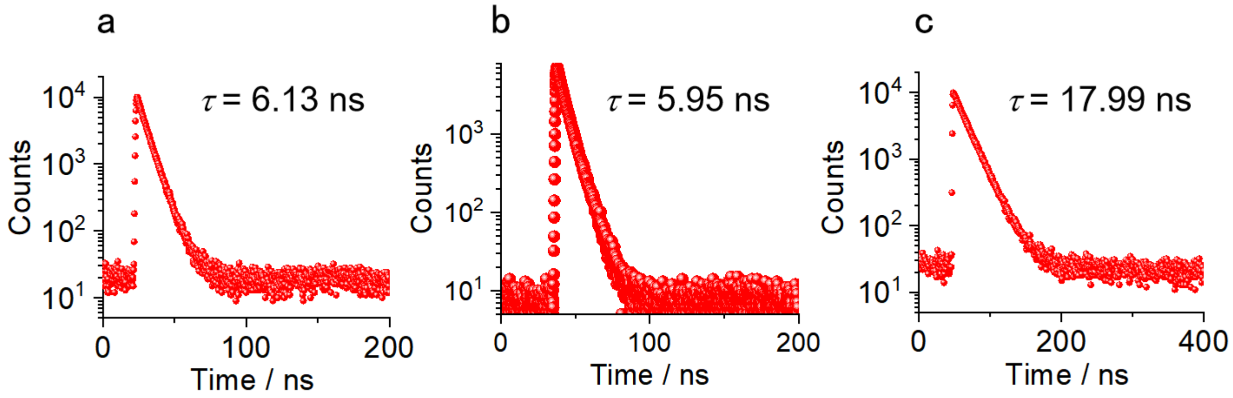

Supplementary Figure 6. Decay curves and lifetimes of crystals of compound 1**.** (a) Straight crystals excited at 540 nm at 298 K. (b) Bent crystals excited at 540 nm at 298 K. (c) Straight crystals excited at 580 nm at 77 K. The lifetimes (τ) were determined by a single-exponential fitting using the function $y = A\exp(-x/\tau) + y_0$.

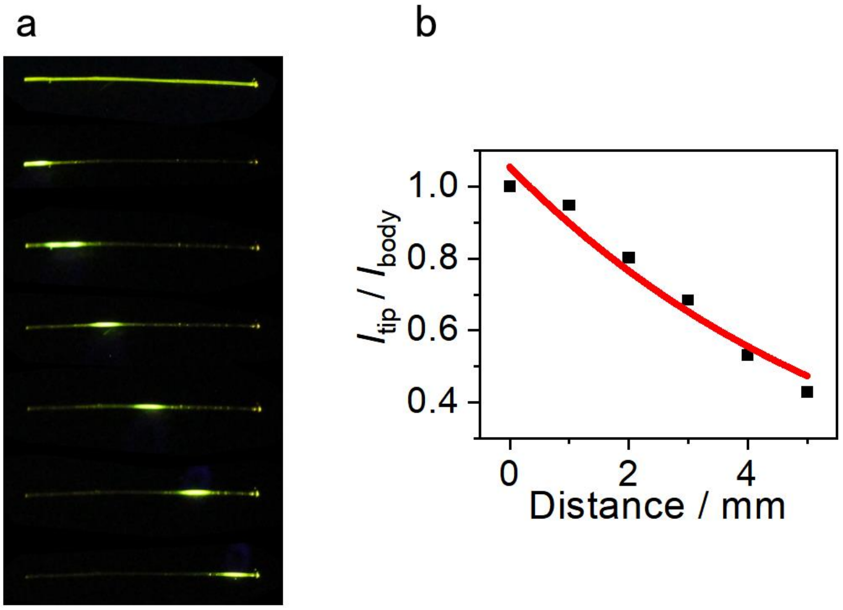

Supplementary Figure 7. Determination of the optical loss coefficient of a straight crystal of compound 1 at 298 K. a) Fluorescence images collected upon excitation of the crystal at different positions. b) The I_{tip}/I_{body} decays of the crystals with a straight shape. The optical loss coefficients (α) reported in the text were determined by a single-exponential fitting of the function $I_{tip}/I_{body} = A\exp(-\alpha D)$, where I_{tip} and I_{body} are the fluorescence intensities of out-coupled and incident light, respectively, D is the distance between the excited site and the end of the crystal for collecting emission, and α is the optical loss coefficient.

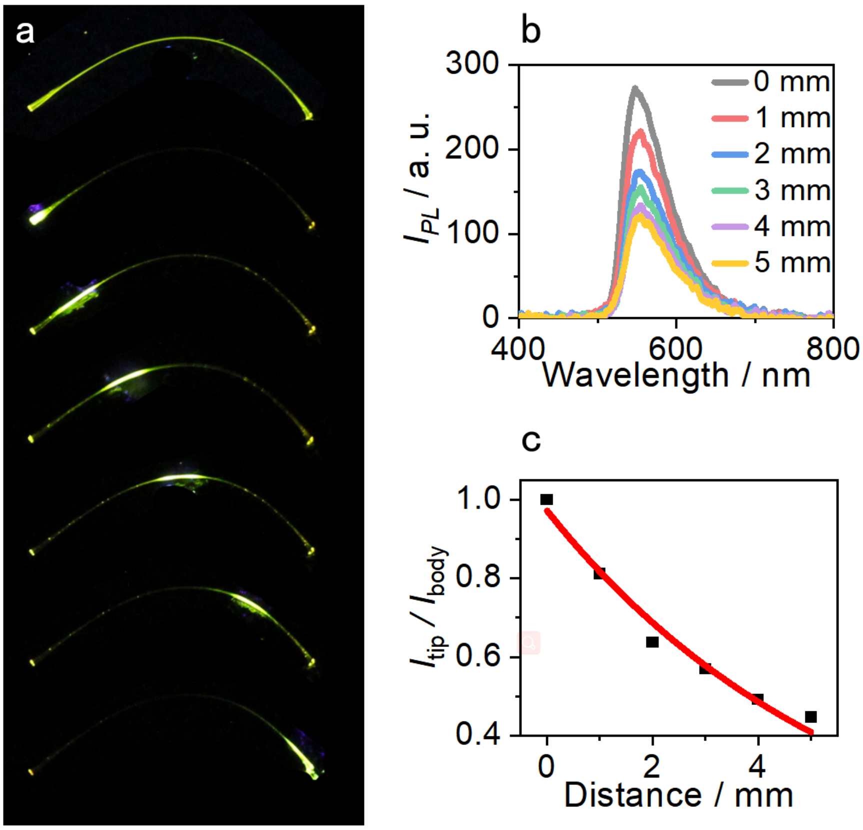

Supplementary Figure 8. Determination of the optical loss coefficient of a bent crystal of compound 1 at 298 K. a) Fluorescence images collected upon excitation of bent crystal at different positions. b) Emission spectra measured at one end of the crystal with various distances (0−5 mm) between the end and the excitation positions. c) The I_{tip}/I_{body} decays of bent crystals at 298 K.

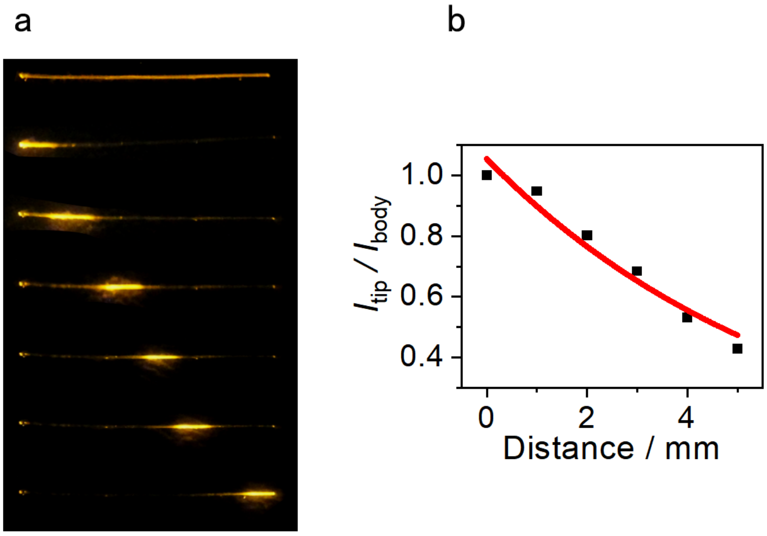

Supplementary Figure 9. Determination of the optical loss coefficient of a straight crystal of compound 1 at 77 K. a) Fluorescence images collected upon excitation of the crystal at different positions (77 K). b) The I_{tip}/I_{body} decays of the crystals with a straight shape at 77 K.

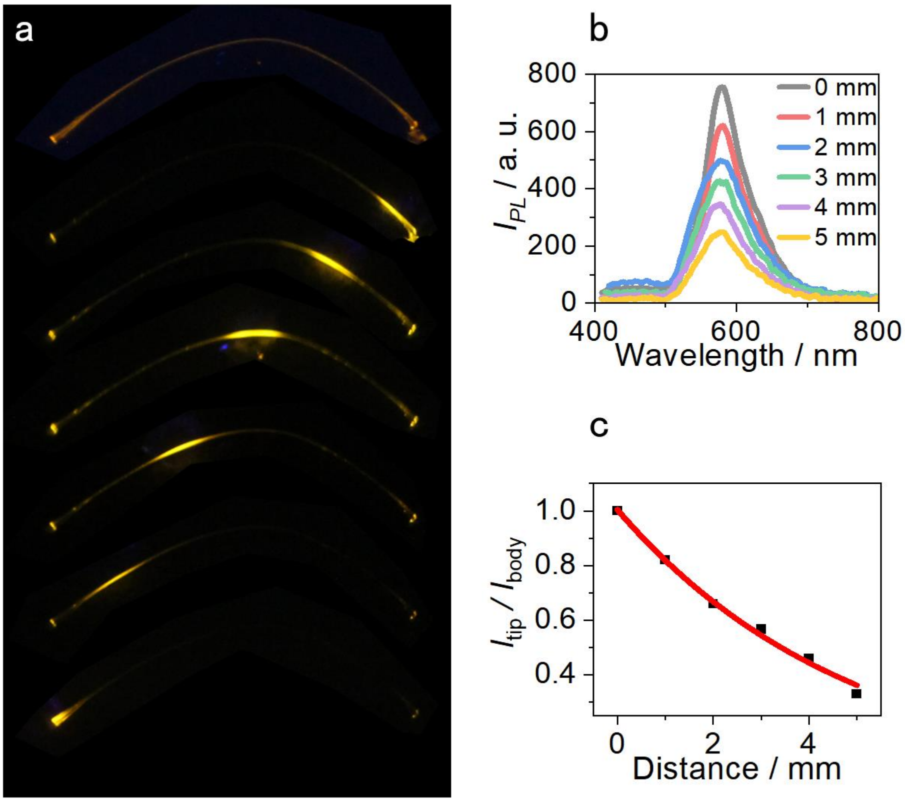

Supplementary Figure 10. Determination of the optical loss coefficient of a bent crystal of compound 1 at 77 K. a) Fluorescence images collected upon excitation of bent crystal at different positions (77 K). b) Emission spectra measured at one end of the crystal with various distances (0−5 mm) between the end and the excitation positions. c) The I_{tip}/I_{body} decays of bent crystals at 77 K.

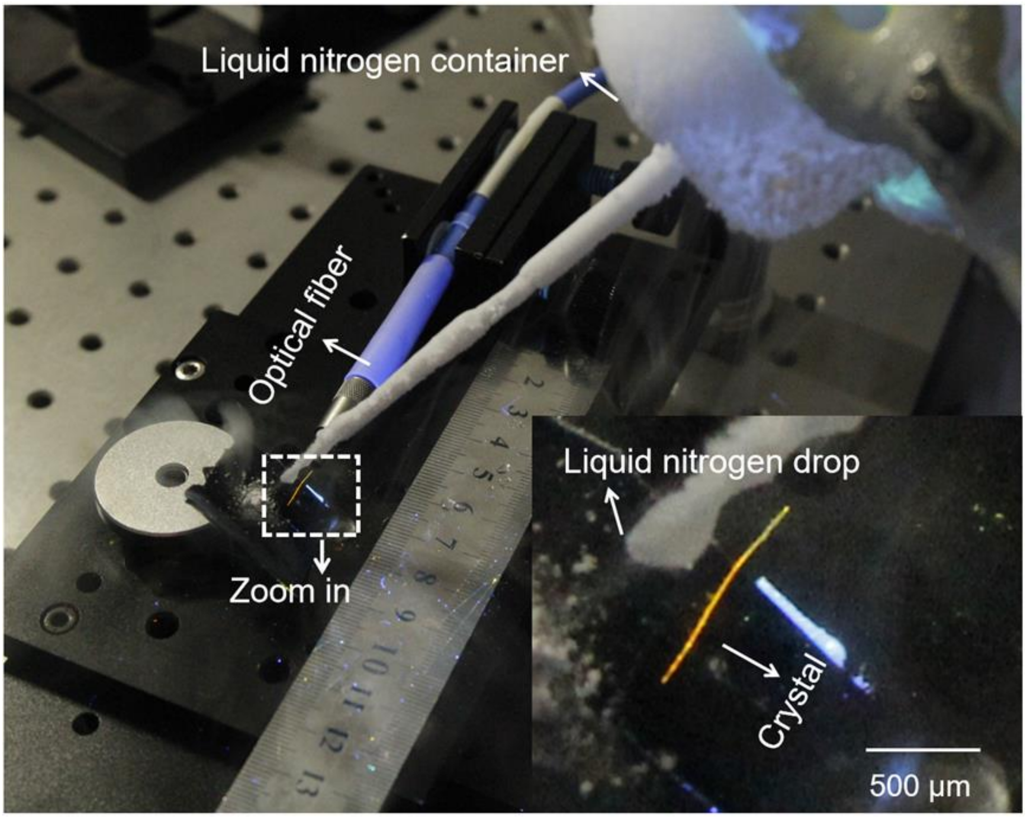

Supplementary Figure 11. Photograph of the experimental setup used for characterization of crystals of compound 1 as optical waveguides at low temperature.

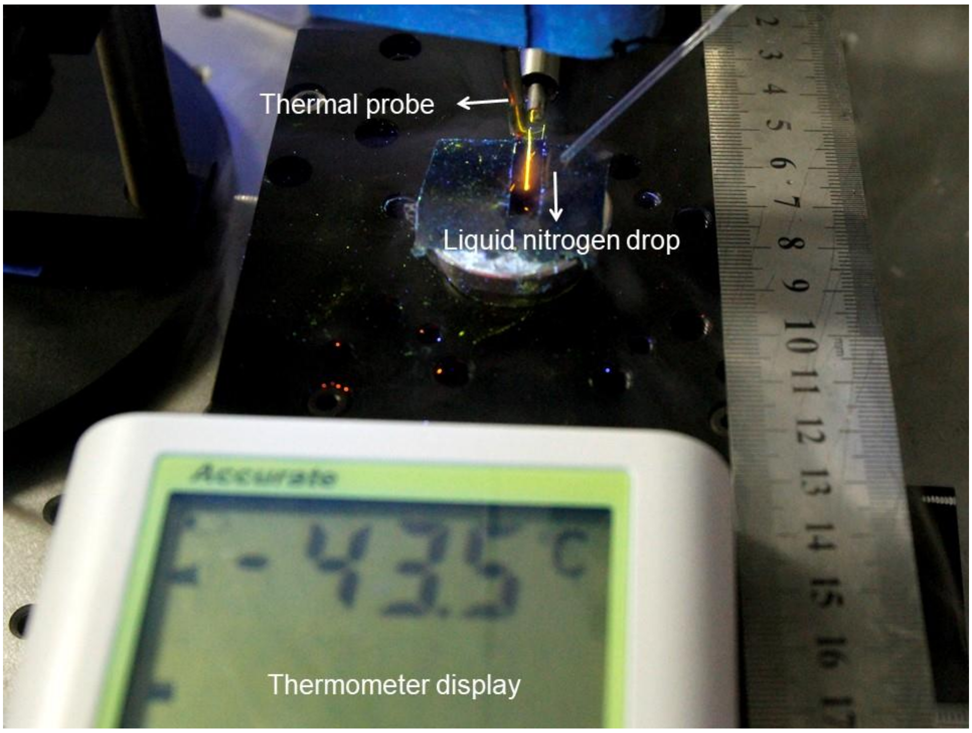

Supplementary Figure 12. Photographs of the experimental setup used for temperature measurement.

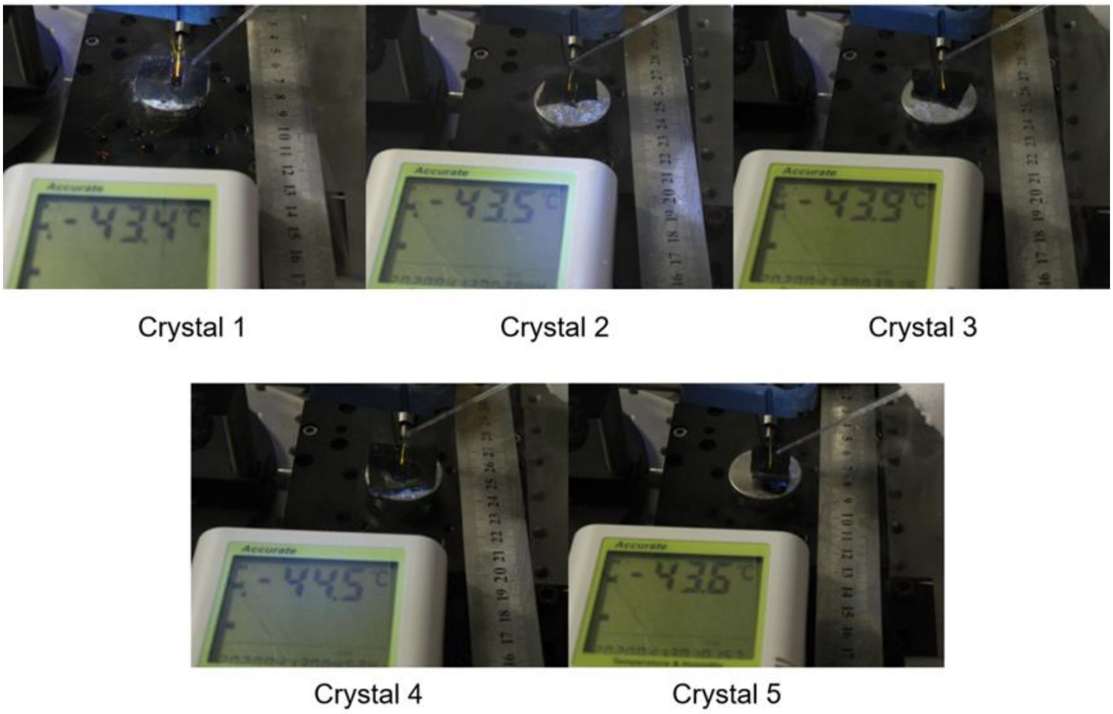

Supplementary Figure 13. Temperature readings for five crystals of compound 1.

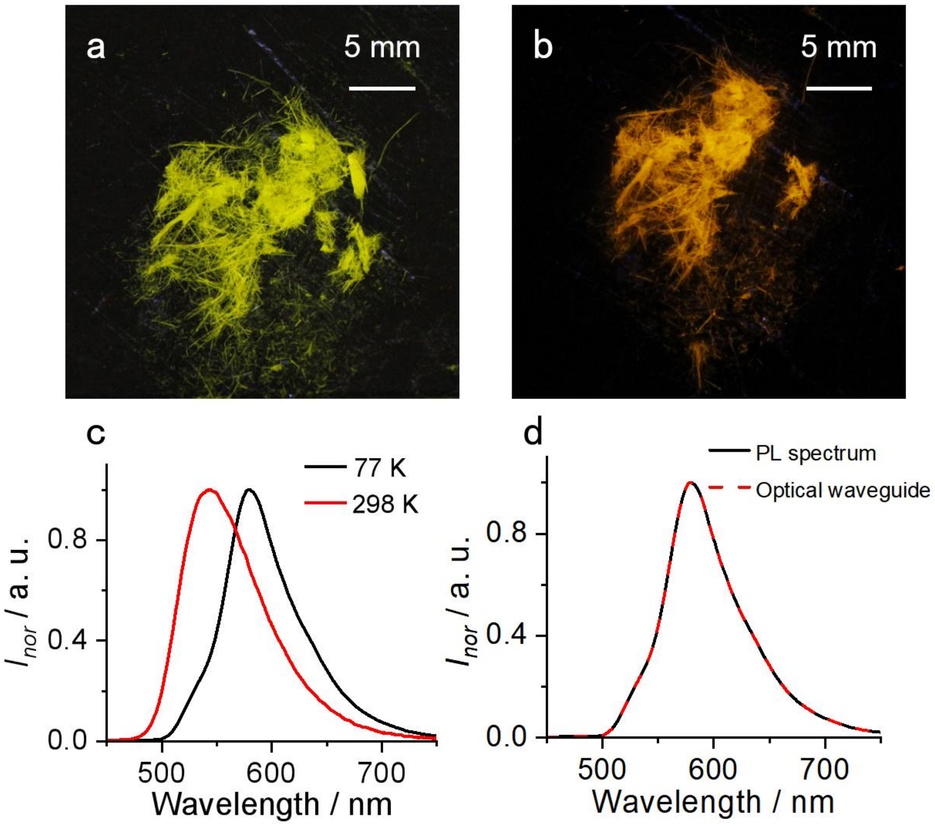

Supplementary Figure 14. Fluorescence at 298 K and 77 K. a) Photograph of crystals of millimeter size at 298 K under UV light. b) Photograph of the crystals of at 77 K under UV light. c) Photoluminescence (PL) spectra of crystals of millimeter size at 298 K and 77 K. d) Overlapped representation of the PL spectra of crystals of millimeter size at 77 K and the optical waveguide signal at 77 K.

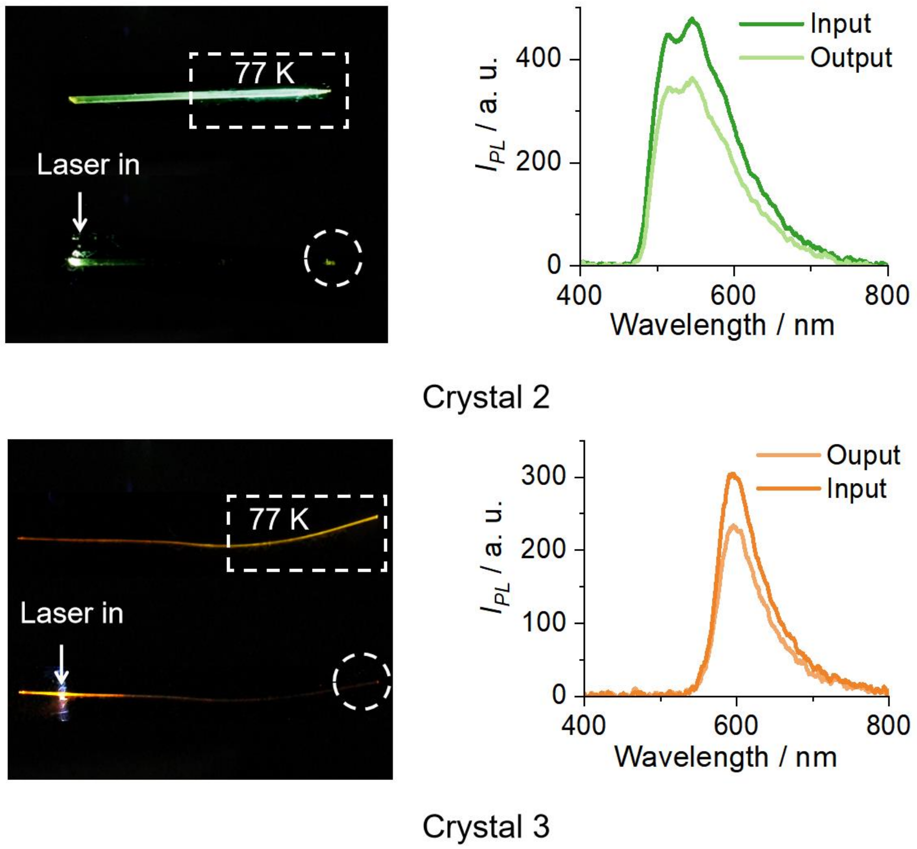

Supplementary Figure 15. Photographs of two other previously reported compounds, compound 2 ("Crystal 2")[1] and compound 3 ("Crystal 3")[2] used as waveguides at low temperature, and the respective emission spectra recorded at the excitation ends and output ends of their crystals.

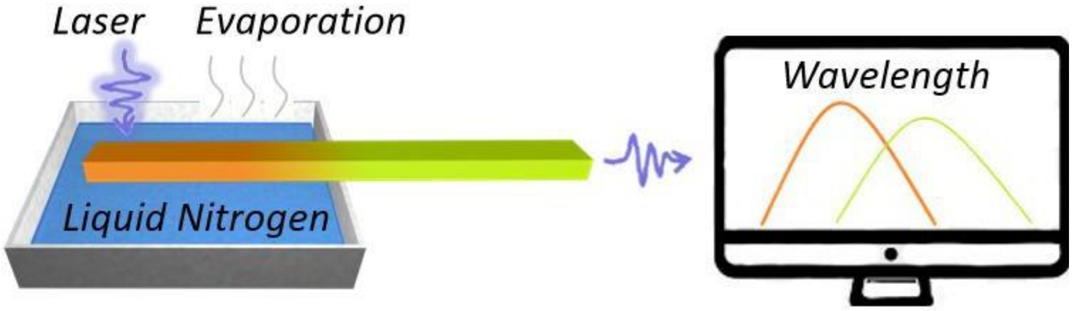

Supplementary Figure 16. Schematic diagram of the experiment used to collect data of the crystals as optical waveguides at different temperatures.

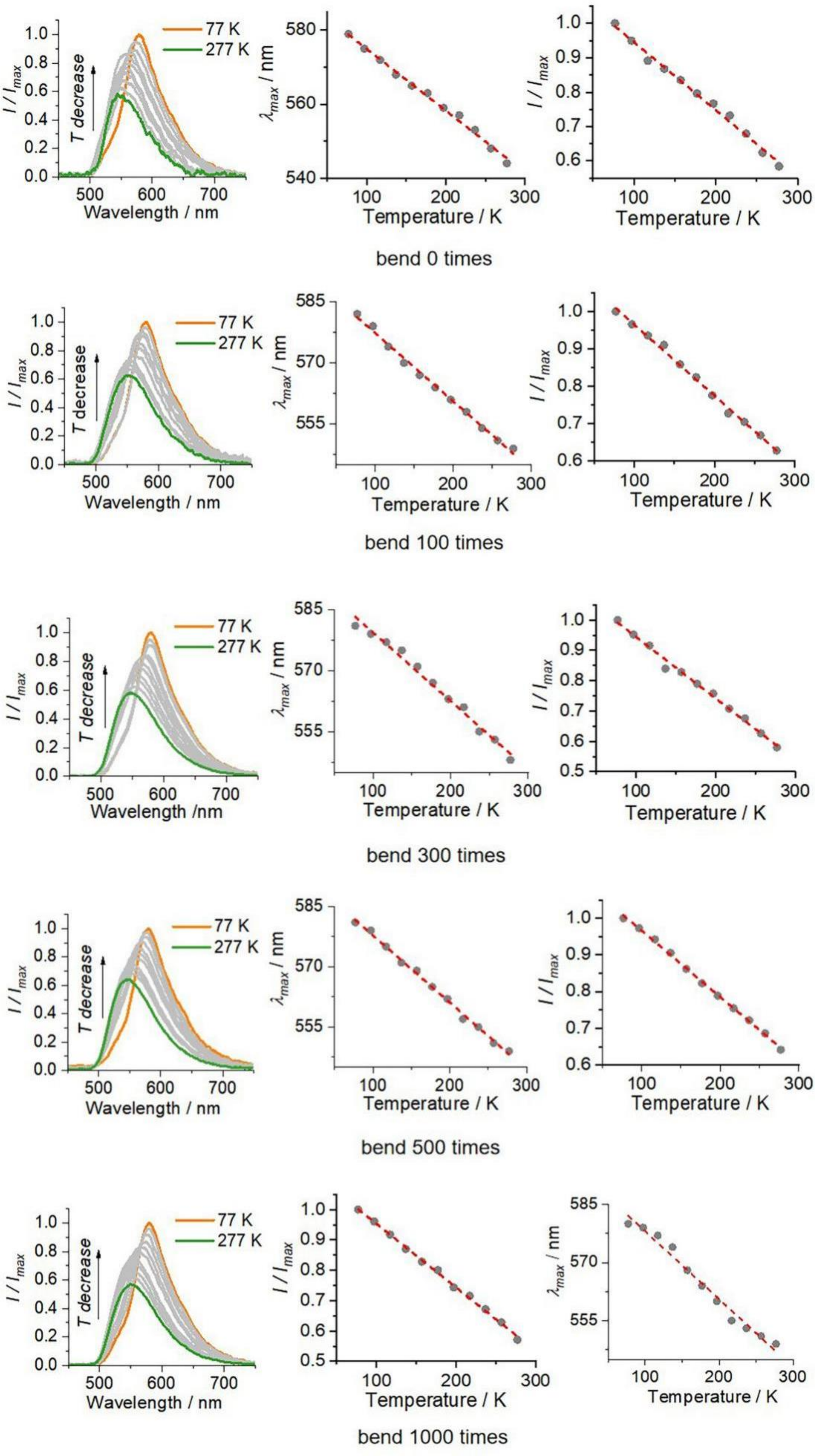

Supplementary Figure 17. Variable-temperature optical waveguide spectra and sensitivity curves of crystal of compound 1 after bending and unbending over different number of times.

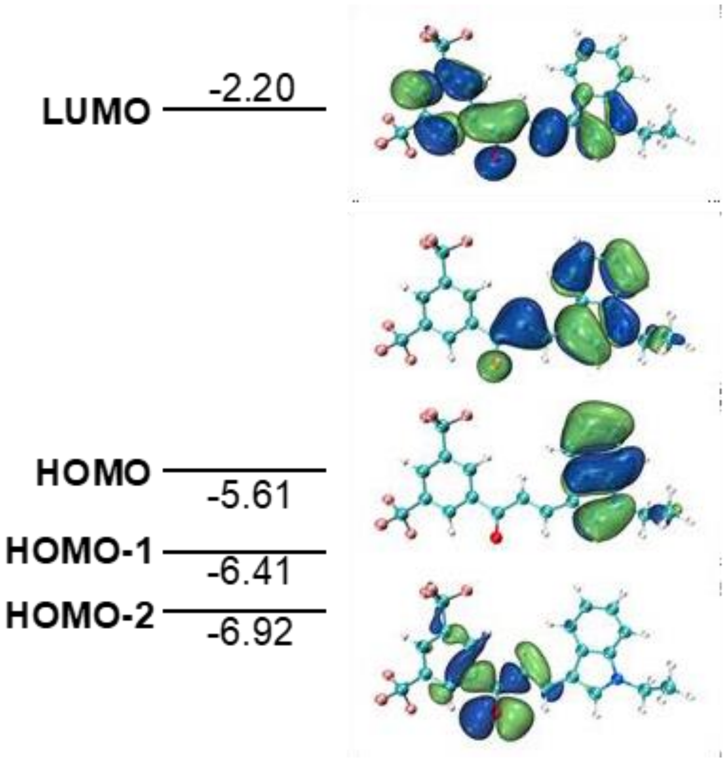

Supplementary Figure 18. Energy levels of HOMO–2, HOMO–1, HOMO and LUMO based on the ground-state geometries of a molecule of compound 1 (the energy is given in electron-volts).

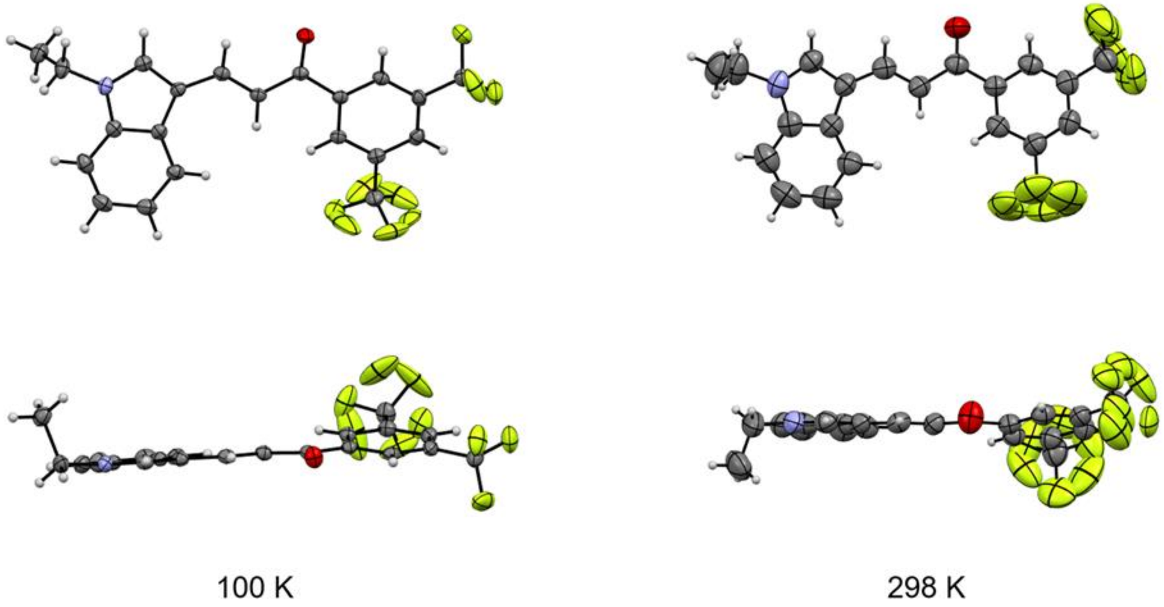

Supplementary Figure 19. ORTEP-style illustration of compound 1 crystal in 100 K and 298 K.

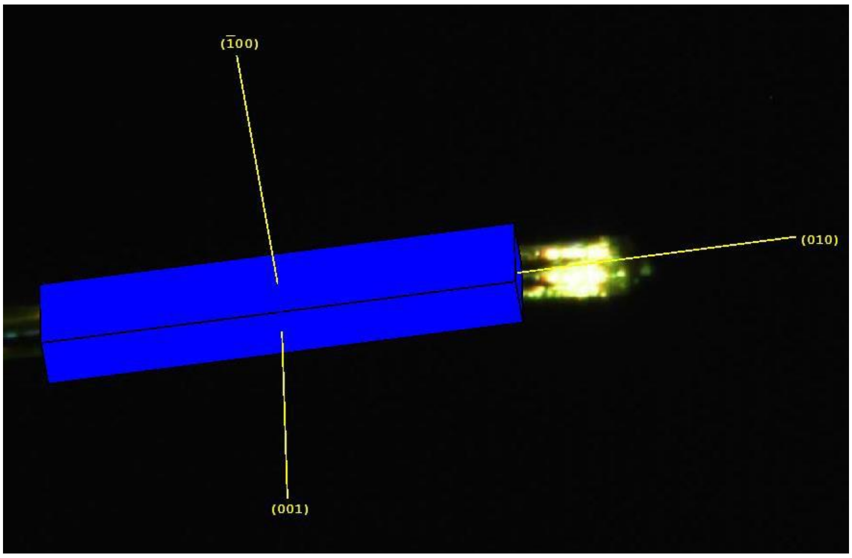

Supplementary Figure 20. Face indexing of a crystal of compound 1 crystal with a typical habit.

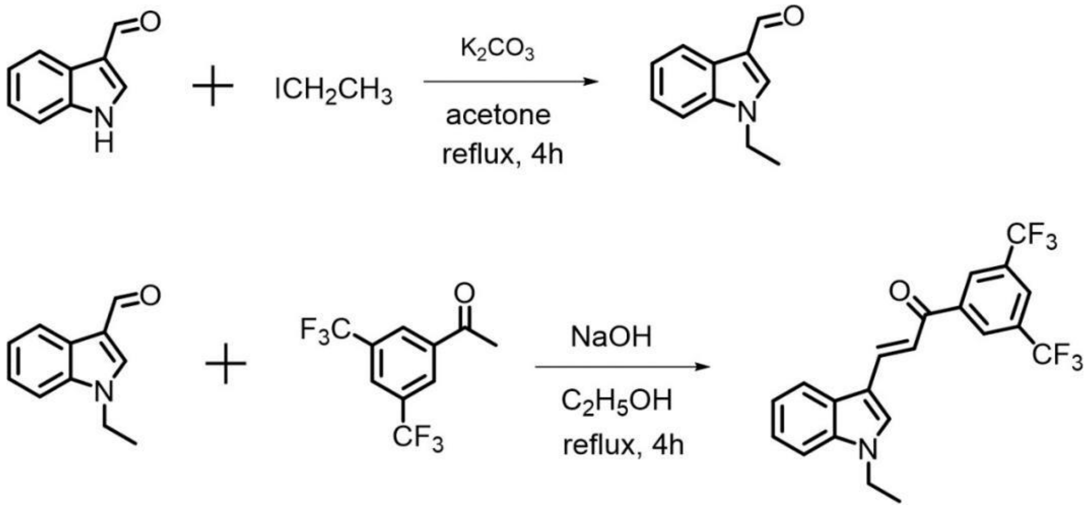

Supplementary Figure 21. Synthetic procedure used to prepare the target compound 1.

Supplementary Tables

Supplementary Table 1. The radiative rate constants and non-radiative rate constants of crystals of compound 1 at 77 K and 298 K

Temperature	Lifetime, τ	PLQY, Y_f^a	Radiative rate constant, K_r	Non-radiative rate constant, K_{nr}
77 K	17.99 ns	0.30	1.67×10^7	3.89×10^7
298 K	6.13 ns	0.16	2.61×10^7	1.37×10^8

[a]PLQY stands for photoluminescence quantum yield.

Supplementary Table 2. Radiative rate constants and non-radiative rate constants of straight and bent crystals of compound 1

Shape of the crystal	Lifetime, τ	PLQY, Y_f^a	Radiative rate constant, K_r	Non-radiative rate constant, K_{nr}
straight	6.13 ns	0.16	2.61×10^7	1.37×10^8
bent	5.95 ns	0.16	2.69×10^7	1.41×10^8

[a]PLQY stands for photoluminescence quantum yield.

Supplementary Table 3. Sensitivity and R^2 obtained by regression analysis for crystals of compound 1 after bending and unbending

Bending cycles	0	100	300	500	1000
R^2 (I / I_{max})	0.9916	0.9954	0.9927	0.9982	0.9977
R^2 (λ_{max})	0.9930	0.9943	0.9849	0.9960	0.9780
Sensitivity (I / I_{max})	−0.0019 /K	−0.0019 /K	−0.0020 /K	−0.0018 /K	−0.0021 /K
Sensitivity (λ_{max})	−0.17 nm /K	−0.17 nm /K	−0.17 nm /K	−0.17 nm /K	−0.17 nm /K

Supplementary Table 4. Calculated excitation energies and oscillator strengths for a molecule of compound 1

	Vertical excitation energy (eV)	Oscillator strength	Vertical emission energy (eV)	Oscillator strength	Adiabatic excitation energy (eV)
S_1	3.08	0.664	2.75	0.783	2.96
S_2	3.41	0.002	2.94	0.000	3.21
S_3	3.67	0.006	2.94	0.297	3.44

Supplementary Table 5. Cartesian coordinates of the optimized molecular structures

State	Coordinates			
S_0	O	-0.52753600	-2.77214300	0.26851100
	N	5.57300000	-1.09959000	-0.08856400
	C	0.78990300	-0.81945000	-0.01883800
	H	0.76232300	0.24292500	-0.21848900
	C	3.82835200	0.35065500	-0.31501300
	C	-1.76659200	-0.76785800	0.08431700
	C	-0.47037000	-1.54419700	0.12587400
	C	3.30564800	-0.98087600	-0.05112800
	C	-1.84439500	0.62186800	0.23264300
	H	-0.95171500	1.21470400	0.38774000
	C	4.42296600	-1.80404500	0.07630200
	H	4.45377600	-2.86710200	0.27434500
	C	5.24481200	0.23454800	-0.32679400
	C	-3.08252300	1.26662100	0.19383800
	C	1.97357200	-1.48609100	0.07085400
	H	1.89585900	-2.55519900	0.26563500
	C	-4.18121100	-0.84713700	-0.14254300
	C	-4.25630200	0.53978000	0.00067400
	H	-5.21284000	1.04597900	-0.04807000
	C	-2.95024200	-1.49592600	-0.09399100
	H	-2.88100100	-2.57180500	-0.20096000
	F	-6.41755900	-0.94124000	-0.91766300
	C	3.26027100	1.61439000	-0.54727000
	H	2.18558900	1.75542500	-0.55042400
	F	-5.94607300	-2.02887400	0.90275600
	F	-5.24568300	-2.77625000	-1.01273300
	C	-5.44678100	-1.64866100	-0.29760000
	F	-2.04098400	3.38462200	-0.01133500
	C	4.09577100	2.70330800	-0.77731500
	H	3.65978000	3.68144700	-0.95598800
	C	6.09024800	1.32316400	-0.55751000
	H	7.16958700	1.21371300	-0.56168400
	C	5.49554200	2.56071600	-0.78359600
	H	6.12210000	3.42815200	-0.96635300
	C	6.92796600	-1.63903700	0.03908300
	H	7.54409600	-1.18483100	-0.74231500
	H	6.87032000	-2.70821900	-0.17870100
	C	-3.15731300	2.75402500	0.41632400
	C	7.53910500	-1.40198500	1.42158800
	H	7.60242200	-0.33467900	1.65026300
	H	8.54988900	-1.81917100	1.45344100
	H	6.94177400	-1.88625200	2.19925400
	F	-4.20844500	3.30744800	-0.22731700
	F	-3.29828400	3.05610700	1.72904200
S_1	O	-0.53376600	-2.81982400	-0.36130000
	N	5.53047900	-1.10249400	-0.00853400
	C	0.80041700	-0.90666500	-0.14715000
	H	0.84907700	0.16127000	0.00158600
	C	3.75512200	0.27002200	-0.38515200
	C	-1.71620200	-0.78729800	-0.08576600
	C	-0.48055400	-1.57436000	-0.20347600

	C	3.27330100	-1.09292900	-0.21971100
	C	-1.76307000	0.61074600	0.06898600
	H	-0.85730600	1.19806200	0.11106000
	C	4.44859000	-1.87309700	-0.00184900
	H	4.51825300	-2.93734700	0.16692400
	C	5.15737000	0.22830300	-0.23226000
	C	-2.97463000	1.26437600	0.16425800
	C	1.97949700	-1.63935500	-0.23888600
	H	1.88336600	-2.71922500	-0.26483000
	C	-4.13943400	-0.80354200	-0.04397300
	C	-4.18621200	0.57471400	0.11327100
	H	-5.13030000	1.09904700	0.18317300
	C	-2.93715300	-1.48097200	-0.14064100
	H	-2.91037300	-2.55497900	-0.26738200
	F	-6.46108400	-0.85363000	-0.48012800
	C	3.15670000	1.48871400	-0.69536100
	H	2.09377200	1.56747900	-0.87489100
	F	-5.74529600	-2.03937400	1.16711200
	F	-5.34965000	-2.66290300	-0.85325800
	C	-5.42184900	-1.58760000	-0.05783400
	F	-1.81585500	3.32674000	0.26540100
	C	3.95565100	2.62431200	-0.79890500
	H	3.49658900	3.57549500	-1.03317800
	C	5.96521200	1.34403600	-0.33821200
	H	7.03939100	1.28861500	-0.21752800
	C	5.33626400	2.55495200	-0.61913800
	H	5.93341200	3.45292300	-0.70757600
	C	6.89641700	-1.54020500	0.26879800
	H	7.55030800	-1.05155800	-0.45382800
	H	6.93184700	-2.61219000	0.08420900
	C	-3.02373200	2.75467100	0.34094400
	C	7.30462200	-1.21268800	1.69915200
	H	7.26316400	-0.13983900	1.88503600
	H	8.32561700	-1.55306900	1.86856500
	H	6.64655500	-1.71609700	2.40695400
	F	-3.79482200	3.34260000	-0.58894200
	F	-3.54379900	3.09997500	1.53195700
S_2	O	-0.53625400	-2.77453600	-0.15147700
	N	5.56284400	-1.05790300	-0.27356000
	C	0.81465300	-0.86654700	-0.11019400
	H	0.80894700	0.21071900	-0.06986300
	C	3.79204500	0.34695700	-0.12738000
	C	-1.75268900	-0.77550600	-0.05375600
	C	-0.48177700	-1.48426400	-0.10387600
	C	3.30665000	-1.01644600	-0.17840100
	C	-1.81714800	0.62860100	-0.03324500
	H	-0.91968700	1.22871000	-0.05254600
	C	4.43290400	-1.81182600	-0.25885200
	H	4.49415900	-2.88864700	-0.31465200
	C	5.20426200	0.27065400	-0.18156800
	C	-3.04226500	1.25919300	0.00377200
	C	1.97740700	-1.56271200	-0.16682400
	H	1.92995400	-2.64688100	-0.20979000
	C	-4.16849100	-0.83667400	-0.00061500
	C	-4.23866800	0.54506000	0.02305300

	H	-5.19168800	1.05661000	0.04503200
	C	-2.95281900	-1.50158400	-0.03570900
	H	-2.93275400	-2.58385200	-0.05617800
	F	-6.50886900	-0.94728100	-0.30021500
	C	3.20262100	1.61650800	-0.04555900
	H	2.13024600	1.74461700	-0.00262000
	F	-5.66206200	-2.12298300	1.29251000
	F	-5.37018000	-2.72162200	-0.75225400
	C	-5.43075700	-1.65417000	0.05544600
	F	-1.93637700	3.33917100	-0.21255100
	C	4.01122200	2.73815300	-0.01563800
	H	3.55749700	3.71872100	0.05108200
	C	6.02475100	1.39709900	-0.15048700
	H	7.10353100	1.31057600	-0.18783900
	C	5.40935500	2.63153900	-0.06791400
	H	6.01413400	3.52893400	-0.04192000
	C	6.92775900	-1.56388200	-0.25662500
	H	7.52542000	-0.94277600	-0.92539400
	H	6.90386700	-2.56806700	-0.67832700
	C	-3.11290000	2.76150200	0.05191900
	C	7.51694800	-1.57724800	1.14725300
	H	7.53938200	-0.57179400	1.56845500
	H	8.53644800	-1.96212100	1.12031800
	H	6.92137000	-2.21329600	1.80247700
	F	-3.99924400	3.24375400	-0.83109700
	F	-3.50205300	3.19937400	1.26023900
S_3	O	-0.62740800	-2.86047300	-0.65881700
	N	5.56118200	-0.95040300	0.09651400
	C	0.80481700	-1.03675000	-0.22396300
	H	0.86018600	0.00259800	0.06582300
	C	3.68839600	0.16642300	-0.50477500
	C	-1.69470200	-0.81348700	-0.15695200
	C	-0.47611300	-1.64421300	-0.37019500
	C	3.28659200	-1.17969600	-0.24085900
	C	-1.68894200	0.57533900	0.04826800
	H	-0.76185000	1.12948900	0.06349800
	C	4.44474800	-1.83095200	0.11556800
	H	4.59997000	-2.86625300	0.37875100
	C	5.13357400	0.25533400	-0.23832000
	C	-2.87143600	1.26681300	0.22297300
	C	1.97235200	-1.75135900	-0.36589700
	H	1.90596600	-2.81924300	-0.53527500
	C	-4.11307500	-0.74812000	-0.00575500
	C	-4.10715300	0.62048600	0.20303400
	H	-5.02796000	1.17173100	0.33838500
	C	-2.93511700	-1.45866800	-0.18595400
	H	-2.94752200	-2.52773200	-0.35626700
	F	-6.47852800	-0.70243900	0.02537400
	C	3.03013700	1.29582200	-1.01548400
	H	1.98732800	1.24907400	-1.29160300
	F	-5.50979700	-2.35027400	1.01852500
	F	-5.53580400	-2.25934200	-1.12877500
	C	-5.40923800	-1.50737300	-0.02372900
	F	-1.63581000	3.28120400	0.37489000
	C	3.74124400	2.45455600	-1.16285800

H	3.25767000	3.33928100	-1.55346900
C	5.84703500	1.47474000	-0.36394500
H	6.89786300	1.54055600	-0.12173400
C	5.14537900	2.54430700	-0.81748100
H	5.63875300	3.49900600	-0.93913500
C	6.89986700	-1.33156100	0.53489500
H	7.60544400	-0.64188700	0.07509400
H	7.09625600	-2.32456300	0.13140000
C	-2.86329900	2.75087200	0.44589100
C	7.01411800	-1.31633900	2.05270800
H	6.82552700	-0.31509500	2.44080300
H	8.01790100	-1.62149100	2.34586100
H	6.29912200	-2.00590400	2.50189500
F	-3.62077000	3.39737100	-0.45705300
F	-3.35915400	3.07903400	1.65235800

Supplementary Table 6. Crystallographic data for a crystal of compound 1 at 100 K and 298 K

Empirical formula	$C_{21}H_{15}F_6NO$	$C_{21}H_{15}F_6NO$
Formula weight	411.34	411.34
Temperature / K	100.0	297.8
Crystal system	monoclinic	monoclinic
Space group	$P2_1/n$	$P2_1/n$
a / Å	12.0224(7)	12.1007(9)
b / Å	4.8149(3)	4.8876(4)
c / Å	31.4650(18)	31.714(2)
α / °	90.00	90.00
β / °	92.271(2)	91.548(3)
γ / °	90.00	90.00
Volume / Å^3	1819.97(19)	1875.0(2)
Z	4	4
Density / g cm^{-3}	1.501	1.457
μ / mm^{-1}	0.134	0.131
F(000)	840.0	840.0
Goodness-of-fit on F^2	1.037	1.026
R_1	0.1100	0.1359
wR_2	0.1531	0.2323
CCDC No.	2155928	2155929

Supplementary References

[1] Pan, X., Zheng, A., Yu, X., Di, Q., Li, L., Duan, P., Ye, K., Naumov, P. & Zhang, H. A low-temperature-resistant flexible organic crystal with circularly polarized luminescence. *Angew. Chem. Int. Ed.* **61**, e2022039 (2022).

[2] Liu, H., Ye, K., Zhang, Z. & Zhang, H. An organic crystal with high elasticity at an ultra-low temperature (77 K) and shapeability at high temperatures. *Angew. Chem. Int. Ed.* **58**, 19081–19086 (2019).
