# Supplementary material for: Fluorescence-based thermal sensing with elastic organic crystals
Source: Nat Commun. 2022 Sep 8;13:5280. doi: 10.1038/s41467-022-32894-w (PMC9458730; doi:10.1038/s41467-022-32894-w)
Supplement: Supplementary file 3 — Description of Additional Supplementary Files [file 41467_2022_32894_MOESM3_ESM.pdf]

File name: Supplementary Data 1

Description: Crystallographic Information File for compound 1 at 100 K.

File name: Supplementary Data 2

Description: Crystallographic Information File for compound 1 at 298 K.

File name: Supplementary Movie 1

Description: Fluorescence of crystal of compound 1 at room temperature and in contact with liquid nitrogen.

File name: Supplementary Movie 2

Description: Repeated elastic bending of a crystal of compound 1.

File name: Supplementary Movie 3

Description: Measurement of the low-temperature optical waveguiding capability of a crystal of compound 1.
